# Transient expression of myofibroblast-like cells in rat rib fracture callus

**DOI:** 10.3109/17453674.2011.652891

**Published:** 2012-02-08

**Authors:** Stuart J McDonald, Philip C Dooley, Aaron C McDonald, Johannes A Schuijers, Alex R Ward, Brian L Grills

**Affiliations:** Tissue and Cell Biology Group, Musculoskeletal Research Centre, La Trobe University, Victoria, Australia

## Abstract

**Background and purpose:**

We have previously shown that early fracture callus of rat rib has viscoelastic and contractile properties resembling those of smooth muscle. The cells responsible for this contractility have been hypothesized to be myofibroblast-like in nature. In soft-tissue healing, force generated by contraction of myofibroblasts promotes healing. Accordingly, we tried to identify myofibroblast-like cells in early fibrous callus.

**Animals and methods:**

Calluses from rat rib fractures were removed 7, 14, and 21 days after fracture and unfractured ribs acted as controls. All tissues were analyzed using qPCR and immunohistochemistry. We analyzed expression of smooth muscle- and myofibroblast-associated genes and proteins including alpha smooth muscle actin (αSMA), non-muscle myosin, fibronectin extra domain A variant (EDA-fibronectin), OB-cadherin, connexin-43, basic calponin (h1CaP), and h-caldesmon.

**Results:**

In calluses at 7 days post-fracture, there were statistically significant increases in expression of αSMA mRNA (2.5 fold), h1CaP mRNA (2.1 fold), EDA-fibronectin mRNA (14 fold), and connexin-43 mRNA (1.8 fold) compared to unfractured ribs, and by 21 days post-fracture mRNA expression in calluses had decreased to levels approaching those in unfractured rib. Immunohistochemistry of 7 day fibrous callus localized calponin, EDA-fibronectin and co-immunolabeling of OB-cadherin and αSMA (thus confirming a myofibroblastic phenotype) within various cell populations.

**Interpretation:**

This study provides further evidence that early rat rib callus is not only smooth muscle-like in nature but also contains a notable population of cells that have a distinct myofibroblastic phenotype. The presence of these cells indicates that in vivo contraction of early callus is a mechanism that may occur in fractures so as to facilitate healing, as it does in soft tissue wound repair.

There is a growing body of evidence to indicate that early, soft fracture callus is smooth muscle-like in nature. The contractile microfilament, alpha smooth muscle actin (αSMA), is abundantly expressed in early fibrous callus and is recognized as a marker of osteoprogenitor cell populations (Kinner et al. 2002, Dooley et al. 2004, Kalajzic et al. 2008). Furthermore, recent findings from our laboratory suggest that this αSMA expression translates into functional smooth muscle-like passive viscoelastic and active contractile properties of early rat rib fracture callus (McDonald et al. 2009, 2011)

We have previously speculated that the cells responsible for such contractile characteristics are myofibroblast-like (McDonald et al. 2011). Myofibroblasts are phenotypically intermediate between smooth muscle cells and fibroblasts, and have a well-documented role in facilitating wound contraction in healing of soft-tissue wounds (Gabbiani 2003, Desmouliere et al. 2005). At around 1 week after soft-tissue injury, fibroblastic stress fibers develop de novo expression of αSMA that enhances contractile force generated by cells (Darby et al. 1990, Hinz et al. 2001, Hinz and Gabbiani 2003). Myofibroblasts have cell-cell and cell-matrix connections that facilitate transmission of this force between cells and to the granulation tissue matrix (Petridou and Masur 1996, Dugina et al. 2001, Hinz et al. 2004).

Around 1 week after injury, new tissue formed during soft tissue healing or fracture healing mainly contains fibrous, granulation-like tissue matrices (McKibbin 1978, Singer and Clark 1999, Mandracchia et al. 2001). It is likely that myofibroblasts would also be abundant in early callus, but expression of αSMA does not conclusively indicate the presence of myofibroblasts (Hinz 2007). A variety of cells express αSMA, and further work using expression and co-expression of other markers is necessary to confirm this hypothesis (Hinz 2007, Eyden 2008).

Although αSMA is the main phenotypic marker of myofibroblasts, these cells also have other recognized intracellular structural proteins, including non-muscle myosin and the smooth muscle protein basic calponin (h1CaP) (Miettinen et al. 1999, Eyden 2008). Myofibroblasts do not contain other smooth muscle-associated proteins such as h-caldesmon (Miettinen et al. 1999, Eyden 2008). Extra domain A splice variant of fibronectin (EDA-fibronectin) is considered to be the best marker of myofibroblastic extracellular matrix, and is necessary for formation and function of myofibroblasts (Tomasek et al. 2002, Hinz 2007). Development of myofibroblastic cell-cell connections is characterized by expression of OB-cadherin and the gap junction protein connexin-43 (Petridou and Masur 1996, Hinz et al. 2004). Despite these characteristics, the only described means of conclusively identifying myofibroblasts by immunohistochemistry is to co-localize both αSMA and OB-cadherin within these cells (Hinz 2007).

If myofibroblast-like cells are present in healing fracture callus, they may have a similar functional role to that described in soft-tissue healing. We have previously hypothesized that osteoprogenitor cells in early, fibrous callus are myofibroblast-like in nature and that their contraction may generate production of tensile forces that favor osteoblastogenesis and thus healing (Nikolovski et al. 2003, McBeath et al. 2004, Woods and Beier 2006). No studies have conclusively detected myofibroblasts in early, fibrous fracture callus. Thus, in the present study we investigated (1) gene expression of smooth muscle- and myofibroblast-associated markers in rat rib callus on days 7, 14, and 21 post-fracture and (2) the location of the protein products of a number of these genes in 7-day callus using immunohistochemistry.

## Methods

### Animals and rib fracture surgery

This project was approved by La Trobe University Animal Ethics Committee (approved December 13, 2007; registration number: AEC 04/11v3). Twenty-eight 16-week-old male Sprague-Dawley rats had their sixth rib fractured as previously described (Dooley et al. 2004). Briefly, they were anesthetized by intraperitoneal injection of a mixture of 20% xylazine and 80% ketamine (0.1 mL/100 g body weight). An incision was made on the lateral aspect of the trunk and the sixth rib was located and then fractured approximately 2 cm from the vertebral column using a pair of fine scissors. The rats were killed with an overdose of CO_2_ 7, 14, or 21 days after fracture.

### qPCR

Unfractured ribs and fracture calluses at 7, 14, and 21 days post-fracture were extracted and stored in RNAlater (Ambion, Austin, TX) at –80°C. RNA was extracted from samples as described previously (McDonald et al. 2011). Reverse transcription was performed using the SuperScript First-Strand Synthesis System according to the manufacturer's instructions (Invitrogen, Mulgrave, Victoria, Australia). Each target gene sequence was located using a PubMed genome sequence search (GenBank). Beacon Designer 2.0 software (Biosoft International, Palo Alto, CA) was used to design optimal forward and reverse primer sequences (Table), which were made commercially (GeneWorks Pty Ltd., Adelaide, SA, Australia). Expression of smooth muscle- and myofibroblast-associated genes in unfractured ribs and calluses was measured using the iCycler iQ Multi-Color Real-Time PCR detection system (Bio-Rad, Hercules, CA) as described previously (McDonald et al. 2011). Using the 2^-ΔΔCT^ (Livak) method, levels of smooth muscle- and myofibroblast-associated gene expression were normalized to that of glyceraldehyde-3-phosphate dehydrogenase (GAPDH) relative to unfractured rib.

**Table:** Primer sequences and melt temperatures (Tm) used in the experiment

Protein encoded NCBI reference by gene			Sequence (5' - 3')	Length(bp)	Tm(°C)
GAPDH	NM_017008.3	Sense	AGTTCAACGGCACAGTCAAGG	21	58
		Antisense	ACATACTCAGCACCAGCATCAC	22	58
aSMA	NM_031004.2	Sense	CCAGCCAGTCGCCATCAG	19	53
		Antisense	GGAGCATCATCACCAGCAAAG	21	56
h1CaP	NM_031747.1	Sense	CAGGCAACTATCAGTCTACAG	21	52
		Antisense	GAGCGTGTCACAGTGTTC	18	50
Connexin-43	AH003191.1	Sense	GCCCTGCCATAAGCCCTCTTG	21	58
		Antisense	GTGACCGCCTGCCTACAACTTC	22	58
Non-muscle	NM_031520.1	Sense	GTGAAGCCTCTCCTCCAAGTG	21	56
myosin		Antisense	TCCGTTCCATCTCCTCAAGTTC	22	55
h-caldesmon	AB049626.1	Sense	TGGAAGCAGAAGAACAAGAAC	21	52
		Antisense	TTCAGCCTCCCTCCTCTC	18	52
EDA-fibronectin	NM019143.1	Sense	GACTGTGTACTCAGAACCCG	20	54
	(nucleotides 5462-5578)	Antisense	ACAGGGTGACCTACTCAAGC	20	54

### Histology and immunohistochemistry

Seven day callus samples were prepared, cryosectioned, and stained as previously described, but with slight modifications (McDonald et al. 2011). For standard histological observation, some sections were stained routinely with 1% toluidine blue. For immunohistochemistry, primary antibodies consisted of a 1:500 dilution of rabbit anti-CaP (recommended for detection of h1CaP and h3CaP) (Santa Cruz Biotechnology Inc., Santa Cruz, CA), a 1:400 dilution of mouse anti-EDA-fibronectin (Santa Cruz), a 1:400 dilution of goat anti-OB-cadherin (Santa Cruz) and a 1:400 dilution of rabbit anti-αSMA (Abcam, Cambridge, UK). Cell nuclei were counterstained with DAPI (indicated by blue fluorescence). Appropriate biotinylated secondary antibody and streptavidin-Cy3 (red fluorescence) or streptavidin-FITC (yellow-green fluorescence) labeling was used (Sigma-Aldrich). A sequential, double stain protocol was used for detecting co-expression of αSMA and OB-cadherin (visualized as orange-brown fluorescence). Immunostained sections were examined under a fluorescent microscope and images were captured as previously described (McDonald et al. 2011). Non-specific controls consisted of incubations omitting primary antibodies.

### Statistics

Non-parametric ANOVA (Kruskal-Wallis) with Dunn's post test was used to assess differences between gene expression ratios in 7, 14, and 21 day callus and in unfractured rib. All data was expressed as mean (SE). A p-value of < 0.05 was considered statistically significant. We used the GraphPad InStat 3 software package for Windows (GraphPad Software Inc., La Jolla, CA).

## Results

### Gene expression

**Figure 1.**
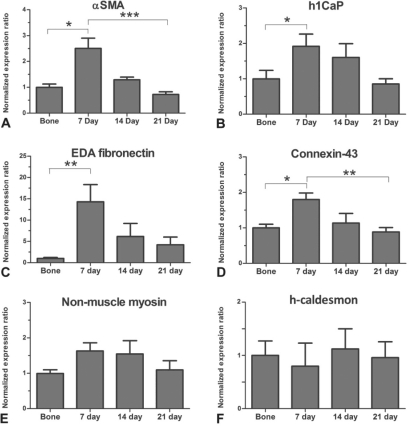
Gene expression in unfractured rib and in 7, 14, and 21 day rib fracture callus of rat (n = 7 biological replicates), normalized to GAPDH relative to normal intact rib. Expression of αSMA mRNA (A), h1CaP mRNA (B), EDA-fibronectin mRNA (C), and connexin-43 mRNA (D) all increased at 7 days post-fracture compared to unfractured rib (*p < 0.05, **p < 0.01). By 21 days post-fracture, expression of both αSMA mRNA and connexin-43 mRNA in callus was less than 7 day expression (***p < 0.001, **p < 0.01, respectively) and was similar to the levels in unfractured rib. The expression of non-muscle myosin mRNA (E) and h-caldesmon mRNA (F) was not significantly different in unfractured rib and callus at any time point.

Mean expression of αSMA, h1CaP, EDA-fibronectin and connexin-43 mRNA was transiently upregulated in healing callus (Figures 1A-D). There was an increase in expression of mRNA encoding αSMA (2.5 fold; p < 0.05), h1CaP (2.1 fold; p < 0.05), EDA-fibronectin (14 fold; p < 0.01), and connexin-43 (1.8 fold; p < 0.05) in 7 day post-fracture callus compared to unfractured rib, and by 21 days post-fracture mRNA expression of these genes in callus had decreased to levels approaching that of unfractured rib (Figures 1A–D). The mRNA expression ratios of non-muscle myosin and h-caldesmon were, however, similar to unfractured rib levels in 7 day callus and remained relatively constant throughout healing (Figures 1E–F).

### Histology and immunohistochemistry

**Figure 2.**
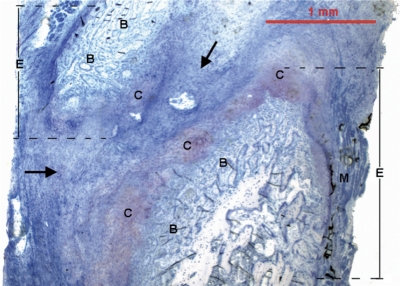
A low-power micrograph of a toluidine blue stained histological section of a rat rib fracture site after 7 days, showing a significant amount of soft-tissue collagenous matrix containing osteoprogenitor cells (arrows) linking together two ends (E) of fractured rib. Immunohistochemical analyses were performed in these fibrous tissue regions. Newly formed cartilage (C) and trabecular bone (B) within the callus are also evident. (M: skeletal muscle). Magnification: ×25.

**Figure 3.**
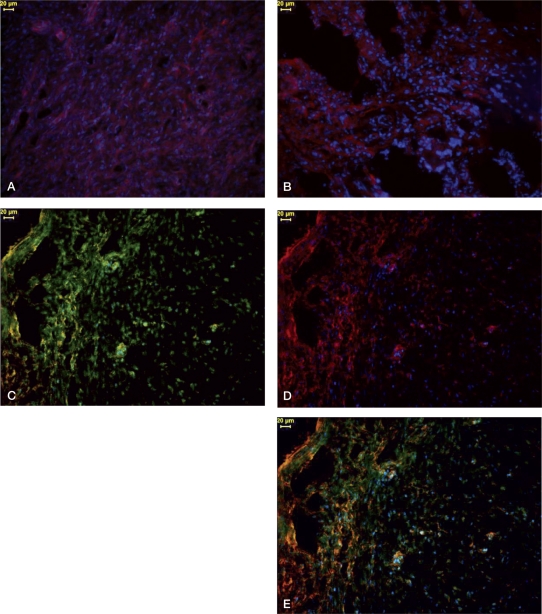
Immunohistochemistry of fibrous callus linking fracture ends in a rat rib fracture after 7 days. Panels C–E show immunostaining of the same callus section. Cell nuclei for panels A–E were stained with DAPI (blue). A.Osteoprogenitor cells show consistent but moderate CaP-LI (indicated by red fluorescence). B.There is intense EDA-fibronectin-like immunoreactivity (LI) (indicated by red fluorescence) in some regions of callus; other areas show only moderate EDA-fibronectin-LI immunostaining. C.OB-cadherin-LI in osteoprogenitor cells (indicated by yellow-green fluorescence). OB-cadherin-LI is particularly intense in high-density fibrous regions (left of picture). Not all cells are OB-cadherin-positive, however. D.Osteoprogenitor cells to the left contain significant αSMA-LI (indicated by red fluorescence), but the cells to the right show only moderate αSMA-LI. E.A sequentially immunostained section for αSMA (red fluorescence) and OB-cadherin (yellow-green fluorescence). Co-localization of these two proteins by immunohistochemistry resulted in a combined brown-orange fluorescence, which is mainly located in osteoprogenitor cells to the left of this micrograph. Note that some cells only show either αSMA-LI or OB-cadherin-LI. Magnification: ×200; scale bars = 20 μm.

Toluidine blue-stained sections of 7 day fracture sites showed large numbers of osteoprogenitor cells within fibrous tissue that linked fracture ends together. Nearer to the ends of fractures, both cartilage and new trabecular bone were present (Figure 2). By immunohistochemistry, we could detect smooth muscle-related and myofibroblast-related proteins but this was variable in osteoprogenitor cells and matrix of fibrous callus 7 days after fracture. CaP-like immunoreactivity (LI) expression was moderate but consistent in most osteoprogenitor cells of callus (Figure 3A). EDA-fibronectin-LI was intense in some regions but it was weaker in other areas within callus (Figure 3B). Intense staining of OB-cadherin-LI and αSMA-LI was evident throughout fibrous callus, but the patterns of immunostaining were different for each (Figures 3C,D). Double immunostaining for OB-cadherin and αSMA revealed that a high proportion of osteoprogenitor cells showed an intense brown-orange fluorescence, which indicated a myofibroblastic characteristic. Some cells only had single immunostaining of either protein (Figure 3E) (results not shown for non-specific control sections).

## Discussion

To our knowledge, this is the first paper to describe a smooth muscle- and myofibroblast-associated gene/protein expression profile in fracture callus at the early stages of fracture healing. We provide evidence that a high proportion of cells responsible for the smooth muscle-like properties of early rat rib callus are myofibroblast-like. Myofibroblasts are known to feature in early healing of soft tissue.

We found upregulation of expression of mRNA for αSMA, h1CaP, EDA-fibronectin and connexin-43, which provides further evidence of the myofibroblastic nature of early callus. It should be noted that these genes are not exclusive to myofibroblasts, but our hypothesis is supported by the fact that myofibroblasts are widely considered not to express the smooth muscle protein heavy (h)-caldesmon (Miettinen et al. 1999, Watanabe et al. 1999, Ceballos et al. 2000). The h-isoform of caldesmon is a smooth muscle-specific protein that inhibits actin-activated myosin ATPase activity (Smolock et al. 2009). Our finding of stable h-caldesmon mRNA expression during fracture healing suggests that the proportion of fully differentiated smooth muscle cells (e.g. those associated with arterioles) remained at levels similar to those in unfractured rib. Furthermore, although a variety of cells—including fibroblasts and myofibroblasts—contain non-muscle myosin, the steady expression of non-muscle myosin during healing is a feature also commonly described in the differentiation of fibroblasts to myofibroblasts (Hinz et al. 2001, Follonier Castella et al. 2010). Taken together, these gene expression results suggest that myofibroblast-associated rather than smooth muscle-associated mRNA expression is transiently upregulated during fracture healing.

Our findings also suggest expression of myofibroblastic marker mRNA translated to protein expression in early callus fibrous regions. Researchers mainly use αSMA as a marker for myofibroblasts; however, this protein is not exclusive to myofibroblasts as both smooth muscle cells and pericytes also express this isoform of actin (Park et al. 1997, Eyden 2008). Myofibroblasts, however, are the only cells that express both OB-cadherin and αSMA (Hinz 2007); thus, co-immunolocalization of these 2 proteins in our experiments provides evidence of myofibroblasts in early callus.

It remains to be seen whether all healing long bone fracture calluses contain myofibroblasts or whether this is unique to healing rib callus. Formation and function of myofibroblasts is dependent on both molecular factors and the local mechanical environment. Specifically, myofibroblasts are considered to work best in environments with high tensile stress (Goffin et al. 2006, Wipff and Hinz 2009). We hypothesize that the constant mechanical stress placed on rib callus during breathing may indeed provide the appropriate mechanical stimuli required for myofibroblastic function. Future investigations will analyze expression of myofibroblastic markers in fractures with different mechanical environments.

We found that transient upregulation in myofibroblastic marker mRNA expression in calluses was followed by a return to unfractured rib levels at 14 and 21 days post-fracture, which suggests that myofibroblasts become reduced in number as callus matures. This finding is consistent with studies on non-fibrotic soft tissue healing where myofibroblasts were thought to disappear by apoptosis at around 16–25 days after injury (Desmouliere et al. 1995). We propose that these cells predominate in early rat rib fracture callus and at this point have a function similar to that seen in soft tissue healing, namely as a tension-generating cell. Having previously shown that early callus is capable of actively contracting ex vivo, we suggest that contraction of callus myofibroblasts in vivo may generate additional tensile forces in callus, similar to those proposed in soft tissue wound healing. Such intracellular tension and fracture matrix tension is thought to favor differentiation of osteoprogenitor cells toward an osteoblastic lineage rather than a chondrocytic one (McBeath et al. 2004, Arnsdorf et al. 2009, Morgan et al. 2010). Thus, a myofibroblast-like osteoprogenitor cell may be a possible therapeutic target for accelerating healing.

Basic calponin (h1Cap) is an actin-associated protein with a minor regulatory role in smooth muscle contraction (Yoshikawa et al. 1998, Matthew et al. 2000). There is also evidence that expression of h1CaP has a non-contractile biological role in regulation of actin cytoskeleton stability (Matthew et al. 2000, Rozenblum and Gimona 2008). We believe that early, contractile callus is likely to be impeded from forming bone due to the presence of smooth muscle proteins such as h1CaP. Mice lacking the gene for h1CaP show increased bone formation and accelerated bone fracture healing (Yoshikawa et al. 1998). Given this information, as well as the data presented in the current experiments, we propose that upregulation of h1CaP in early, contractile callus is likely to inhibit callus bone formation and that the subsequent downregulation of h1CaP in callus over the ensuing weeks may thereby facilitate osteogenesis of callus.

In conclusion, our study provides evidence that myofibroblast-like cells are present in healing rat rib fracture callus. Transient upregulation of expression of genes encoding certain myofibroblastic makers, together with prominent immunostaining for myofibroblasts in callus fibrous regions, suggests that a population of early callus osteoprogenitor cells are myofibroblast-like in nature. Thus, these cells possibly have a role in fracture healing that is similar to what they have in soft tissue wound healing.
